# Withdrawn Behavior in Preschool: Implications for Emotion Knowledge and Broader Emotional Competence

**DOI:** 10.3389/fpsyg.2022.895557

**Published:** 2022-06-30

**Authors:** Samantha E. Clark, Robin L. Locke, Sophia L. Baxendale, Ronald Seifer

**Affiliations:** ^1^Department of Psychology, University of Massachusetts Dartmouth, Dartmouth, MA, United States; ^2^Frank Porter Graham Child Development Institute, The University of North Carolina at Chapel Hill, Chapel Hill, NC, United States; ^3^Department of Psychiatry and Human Behavior, Alpert Medical School of Brown University, Providence, RI, United States; ^4^Bradley/Hasbro Children’s Research Center, E.P. Bradley Hospital, East Providence, RI, United States

**Keywords:** preschoolers, emotion, development, emotion knowledge, context, anger, withdraw, language

## Abstract

The present study investigated the respective roles of withdrawal, language, and context-inappropriate (CI) anger in the development of emotion knowledge (EK) among a subsample of 4 and 5 year-old preschoolers (*n* = 74). Measures included parent-reported withdrawn behavior, externalizing behavior, and CI anger, as well as child assessments of receptive language and EK. Ultimately, findings demonstrated that receptive language mediated the relationship between withdrawn behavior and situational EK. However, CI anger significantly interacted with receptive language, and, when incorporated into a second-stage moderated mediation analysis, moderate levels of CI anger rendered the indirect effect of withdrawn behavior on situational EK via receptive language insignificant. Cumulatively, these findings demonstrate a mechanism by which withdrawal may impact EK. They also indicate that such an effect may be attenuated in children with moderate levels of CI anger. Implications of these findings are discussed.

## Introduction

Upon entering preschool, many children are already quite adept in understanding, expressing, and managing their own emotions, as well as discerning the emotions of others (Saarni, 1990; Dunn, 1994; Denham, 1998). These three core components of emotional competence (i.e., emotion knowledge, emotional expressiveness, and emotion regulation; Saarni, 1999) enable a child to effectively navigate their increasingly complex social world and to form meaningful peer bonds (Howes, 1987; Parker and Gottman, 1989; Saarni, 1990; Parke, 1994; Denham et al., 2003). Consequently, emotional competence can be considered integral to social competence (Halberstadt et al., 2001; Denham et al., 2003). It is not surprising, then, that poor functioning in any of these areas of emotional competence is associated with increased risk for poor social functioning (Eisenberg et al., 1993; Miller and Olson, 2000; Miller et al., 2005; Garner and Lemerise, 2007; Locke et al., 2015) and psychological maladjustment (Denham et al., 2002; Heinze et al., 2015; Locke and Lang, 2016).

### Associations Between Emotion Knowledge and Social and Behavioral Functioning

Of particular significance to emotional competence is emotion knowledge, which comprises a broad range of skills necessary to recognize emotion expressions and to interpret social contexts and behavioral cues (Denham, 1998). Children adept in these skills are better able to regulate their own emotions (Barrett et al., 2001; Eisenberg et al., 2005; Miller et al., 2006; Hudson and Jacques, 2014). They are also far more socially competent. For instance, preschoolers with high emotion knowledge are rated as more likeable by peers, receive more positive nominations from teachers, and are more likely to succeed in school (Izard et al., 2001; Denham et al., 2003, 2012, 2015; Trentacosta and Izard, 2007). Moreover, poor emotion knowledge has consistently been shown to predict internalizing behaviors (e.g., anxiety, depression, and social isolation) and externalizing behaviors (e.g., aggression, hyperactivity, and conduct problems; for review, see Trentacosta and Fine, 2010), both of which come with their own set of negative implications for social competence (Bongers et al., 2008; Bierman et al., 2015; Heinze et al., 2015) and long-term psychological wellbeing (Farrington, 1989; Moffitt, 1993; Betz, 1995; Liu et al., 2011; Thompson et al., 2011). Given these far-reaching consequences, it is imperative to improve upon current understanding of the factors which contribute to poor emotion knowledge and, in turn, decreased emotional and social competence.

### The Role of Withdrawn Behavior in Emotion Knowledge Development

Behavioral withdrawal is one factor which may impede upon the development of emotion knowledge, as it often includes decreased environmental interaction, which is vital for normative social, emotional, and cognitive development (Rubin et al., 1998). In regard to emotional development, withdrawn children may learn less information about emotions due to disengagement from or avoidance of social situations. More precisely, decreased social interaction may translate into delays in developing relations between facial expressions, situational activators, and emotion-eliciting experiences (Schultz et al., 2001; Trentacosta and Fine, 2010). The quality of such interactions may also be compromised (McClure and Nowicki, 2001). For instance, social deficits (e.g., gaze aversion) may decrease a child’s ability to gather emotion-related cues from their environment (McClure and Nowicki, 2001). Taken together, these effects of withdrawn behavior may have profound consequences for emotion knowledge.

### The Intermediary Role of Language in the Withdrawal-Emotion Knowledge Link

In particular, withdrawn behavior may impact emotion knowledge by disrupting normative language development. With fewer peer interactions, withdrawn children may miss experiences necessary for reaching language milestones (Guedeney et al., 2016). This is a reasonable assertion given that social interaction is considered by many to be central to language development (Kuhl, 2007; Bruce and Hansson, 2011). Decreased peer interactions, then, may provide one explanation for the commonly observed negative association between childhood withdrawal and both receptive and expressive language (Paul and Kellogg, 1997; Cohen and Mendez, 2009; Spere et al., 2009; Smith-Watts et al., 2014; Curby et al., 2015). This delayed language development may, in turn, have a negative influence on the development of emotion knowledge. According to Conceptual Act Theory (CAT; Barrett, 2006), language not only scaffolds emotion concept knowledge but also allows individuals to later use such concept knowledge to make sense of sensory perceptions in specific contexts (Lindquist et al., 2015). Evidence of a positive association between language and emotion knowledge (Cutting and Dunn, 1999; Schultz et al., 2001; Miller et al., 2006) supports this emotion knowledge-language link proposed by CAT. Cumulatively, these associative ties between withdrawn behavior, language, and emotion knowledge suggest that language deficits would be a likely mediator for poorer emotion knowledge in withdrawn children.

### Context-Inappropriate Anger and Its Relevance to Withdrawn Behavior

If withdrawn children are more prone to difficulties in understanding the emotional context, they may also struggle to regulate emotions in an adaptive, situationally responsive way (Thompson, 2001; Miller et al., 2006). For instance, they may show affect which is “context-inappropriate (CI; Locke et al., 2009),” such as responding with anger in positive, rewarding situations. Previous studies have demonstrated that children who display CI anger do have worse emotion knowledge than their peers (Locke et al., 2015; Locke and Lang, 2016). Perhaps then, a tendency to behaviorally withdraw would be associated with CI anger responses. In such cases (i.e., where children show both behavioral withdrawal and CI anger), withdrawal may negatively impact emotion knowledge through deficits in language (as we generally suspect for withdrawn children). Alternatively, children who show CI anger may both withdraw and have poorer emotion knowledge because they are not motivated to engage with affective cues in their environment.

This latter explanation may prove viable given that children who show CI anger have decreased hypothalamic-pituitary-adrenal (HPA) axis activity (as indicated by their lower basal morning cortisol levels; Locke et al., 2009), and low cortisol (or hypocortisolism) may serve to impede arousal and engagement with one’s environment (Adam and Gunnar, 2001; Gunnar and Vazquez, 2001). Hypocortisolism has also been implicated in self-regulatory behaviors and executive functions (Blair et al., 2005) that may impact emotion knowledge (Schultz et al., 2001; Denham et al., 2012), above and beyond language ability (von Salisch et al., 2015). Thus, the limited attentional and behavioral control abilities inherent to poor self-regulation may make it difficult for children to learn about emotions through social relationships (Schultz et al., 2001). Considering CI anger’s associations with low cortisol (Locke et al., 2009), attention difficulties (Locke and Lang, 2016), and externalizing behavior (Locke et al., 2015, 2017), poor executive function is likely present in these children. Thus, even with adequate language skills, withdrawn children showing CI anger may still have poorer emotion knowledge than their agemates.

Taken together, these studies suggest that there may be multiple pathways leading to poor emotion knowledge in early childhood. They also highlight various existent gaps in the literature, which upon exploration may serve to further elucidate understanding of this critical component of emotional competence. Regarding the relationship between withdrawn behavior and language, many studies have evaluated the effect of language impairments/deficits on withdrawn behavior, but only a few (e.g., Redmond and Rice, 1998) have considered if withdrawn behavior may impact language, particularly when this construct is broadened to include forms of withdrawal not related to fearfulness/avoidance. Furthermore, to our knowledge, the ability of language to mediate the effect of withdrawn behavior on emotion knowledge has not been evaluated. Secondly, though CI anger has been demonstrated to predict emotion knowledge (Locke et al., 2015; Locke and Lang, 2016), its relationship to withdrawn behavior has not been considered. Finally, exploration of how CI anger impacts the proposed mediation effect is warranted, as other factors (e.g., poor executive function and self-regulation; Blair et al., 2005; Willcutt et al., 2005) may be more relevant than language ability for proper acquisition of emotion knowledge in withdrawn children who show CI anger.

### The Present Study

The objective of the present cross-sectional study was to assess how withdrawn behavior, receptive language, and CI anger interplayed to influence emotion knowledge in a non-clinical, community-based sample of preschoolers. Our primary aim was to determine if withdrawn behavior predicted emotion knowledge and if language acted as a mediator in this effect. We expected children’s receptive language would mediate the association between withdrawn behavior and situation EK (but not recognition EK; hypothesis one), as the former requires higher-level cognitive abilities (Denham and Couchoud, 1990; Widen and Russell, 2010; Bassett et al., 2012) and should, thus, be more vulnerable to decreased peer interaction and language delays (Kuhl, 2007; Bruce and Hansson, 2011; Guedeney et al., 2016). Our secondary aim was to assess associations among CI anger and both withdrawn and externalizing behavior. This aim allowed us to attempt replication of the positive association between externalizing behavior and CI anger noted in previous studies (Locke et al., 2015, 2017; Locke and Lang, 2016); it also allowed us to explore novel associations between CI anger and withdrawn behavior. Here, we expected that children who expressed greater CI anger would show more externalizing behavior (hypothesis two). We also expected such children to be more withdrawn than other children (hypothesis three), in part, perhaps because of their inherent hypoarousal and the resultant disengagement with their environment.

**FIGURE 1:**
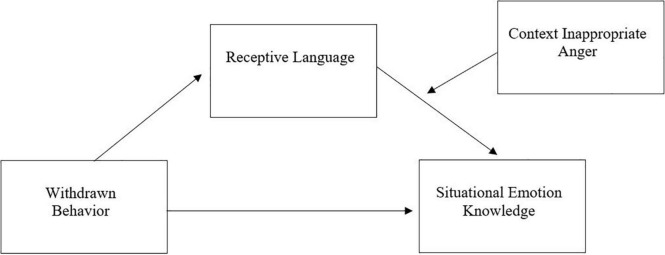
Conceptual model of moderated mediation by CI anger.

Finally, a tertiary aim of this study was to test whether CI anger moderated the relationship between language and emotion knowledge and, further, if our predicted mediation effect of language on the relationship between withdrawn behavior and emotion knowledge would be moderated by CI anger. We expected CI anger would moderate the relationship between receptive language and situational emotion knowledge (hypothesis four). If factors other than language are more pertinent to emotion knowledge development in children who show CI anger, then increasing levels of CI anger may diminish the ability of language to predict situational emotion knowledge. Finally, we expected CI anger would moderate the mediating effect of language on the association between withdrawn behavior and emotion knowledge (hypothesis five). If language ability has less impact on emotion knowledge, then language may not mediate proper emotion knowledge acquisition for withdrawn children showing CI anger. Such children may instead have more salient cognitive deficits (e.g., poor executive function) and affective deficits (e.g., disengagement) that hinder acquisition of emotion knowledge. Consequently, we expected that children who show CI anger may possess attributes which can operate independently of language to impair a child’s ability to understand contextual cues surrounding emotion. For a conceptual model of the proposed moderated mediation analyses based on Hayes (2018), see Figure 1.

## Materials and Methods

### Participants

Participants were 74 4 and 5 year-old preschoolers (*M* = 55 months, *SD* = 6 months; 49% male) from the northeast region of the United States. The community-based, non-clinical sample equally recruited from local public and private preschools was reflective of local demographics (66.7% Caucasian, 6.7% African-American, 1.7% Asian, 3.3% other race, 21.7% multiracial; 16.3% Hispanic/Latino) and economically diverse (annual income: *M* = $41,190, *SD* = $37,768; $3,600 ≤ *x* ≤ $170,000; 52% <poverty level). In prior research, CI anger was not shown by a majority of children (Locke et al., 2009, 2015; Locke and Lang, 2016). Therefore, a portion of the study sample of 74 children (42%) was recruited from a larger screening sample of 149 children based on parent-reported CI anger (described in greater detail within “Materials and Methods”). Overall, the study sample had lower receptive language (*M* = 99.88, *SD* = 13.61) than the larger screening sample (*M* = 105.86, *SD* = 13.07), *t*(140) = 2.67, *p* = 0.01, but the two samples did not differ on externalizing behavior: *t*(124) = 0.10, *p* = 0.92; withdrawn behavior: *t*(124) = 0.18, *p* = 0.86; or CI anger: *t*(127) = 0.38, *p* = 0.71. Parents provided written informed consent, and all procedures were approved by the University Institutional Review Board.

### Procedure

The subsample of 74 children participated in a larger study of emotion, which entailed additional child assessments completed across multiple days at their preschool. Relevant to this study, children were individually interviewed with age-appropriate measures that assessed emotion knowledge and receptive language. Each of these interviews took approximately 20 min to administer. On separate days prior to the emotion knowledge and receptive language interviews, children’s teachers and parents also completed questionnaires to assess child functioning. Families were compensated $40.00, and teachers were compensated $10.00 for their participation.

### Measures

#### Context-Inappropriate Anger

Parents completed an 80-item questionnaire on children’s affect. Three items assessed context-inappropriate anger (i.e., “Sometimes becomes angry for no apparent reason”; “Gets mad about things more than other children”; “In situations where most children become sad, afraid, or anxious (for example, when lost at the mall), my child becomes angry”; Locke and Lang, 2016). The remaining items assessed context-inappropriate emotional responses (Locke and Lang, 2016; Child Behavior Questionnaire (CBQ), Rothbart et al., 2001) or randomly selected items from multiple CBQ subscales and were included to reduce respondents’ awareness of the study’s hypotheses. Following CBQ format, items were rated on a 7-point rating scale (1: extremely untrue of your child to 7: extremely true of your child). An average of the three CI anger items (1 ≤ *x* ≤ 7) was used in analyses (α = 0.73). Scores on this measure of CI anger have been associated with attention problems and emotion knowledge in preschool-aged children (Locke and Lang, 2016) and physiology and externalizing behavior in middle childhood (Locke et al., 2017). At least one of the three items of CI anger was endorsed for 28% of the children, which corresponds with frequency of CI anger observed in prior studies (Locke et al., 2009, 2015; Locke and Lang, 2016).

#### Language Skills

Child receptive language was assessed using the Peabody Picture Vocabulary Test (PPVT-4; Dunn and Dunn, 2007). The PPVT, which yields standardized scores, exhibits good test-retest reliability (α = 0.91, −0.97) and low item bias; it has also been shown to correlate with other measures of vocabulary and general intelligence (Bracken and Murray, 1984; Reynolds et al., 1984).

#### Emotion Knowledge

Emotion knowledge was assessed using portions of several existing instruments, each of which has been used with preschoolers [Affect Knowledge Test (AKT), Denham, 1986; Emotion Matching Test (EMT), Izard et al., 2003; Morgan et al., 2010], and a portion of the Knowledge Assessment Interview (KAI; Kusché et al., 1988). Scores on these measures correlate with naturalistic observations of children’s emotional competence [i.e., school free play (Denham, 1986; Morgan et al., 2010)] and could be useful indicators of change implemented by socioemotional curricula such as the PATHS and Emotions Course programs (Morgan et al., 2010). Our comprehensive assessment included the following measures of recognition EK, expressive EK, and behavioral situational EK:

##### Recognition Emotion Knowledge

We assessed children’s ability to recognize faces depicting four discrete emotions (Happy, Sad, Angry, and Scared) using the EMT. Children were presented with a photo of a child posing a discrete emotional expression and asked to indicate “which child feels the same way” from a panel of four photos of children posing emotional expressions. From this task, a total score indicated the number of emotions correctly identified out of 10 (1 ≤ *x* ≤ 10). Children were next asked to indicate which child in a different panel of four children felt a given emotion (e.g., “show me the one who feels happy”). A total score indicated the number of emotions correctly identified out of 12 (1 ≤ *x* ≤ 12). Lastly, using four drawn feeling faces from the AKT, children were asked to identify the face that depicted the stated emotion. Following Denham (1986), responses for the four items were scored 0 if incorrect, 1 if only the correct valence was given (e.g., “sad” for “angry”), and 2 if the correct discrete emotion was identified. A total score was computed from the average across these items (1 ≤ *x* ≤ 8).

##### Expressive Emotion Knowledge

Children viewed EMT photos and were asked to state the emotion that the child was feeling. A total score was computed to indicate the number of emotions correctly labeled out of eight (1 ≤ *x* ≤ 8). In another task, the four drawn feeling faces from the AKT were presented, and children were asked to identify each emotion using words. Responses were scored in the same manner as the recognition AKT task (1 ≤ *x* ≤ 8). Children were also asked to “name all the different feeling words you can think of,” based on the KAI. A total score indicated the number of emotion words the child generated (1 ≤ *x* ≤ 6). As emotion recognition knowledge and expressive EK were significantly correlated [*r*(72) = 0.50, *p* < 0.001], we computed a mean of the six standardized Recognition and Expressive EK scores to indicate *Recognition/Expressive EK* (α = 0.70).

##### Behavioral Situational Emotion Knowledge

Finally, we assessed children’s ability to understand the emotions of others via behavioral cues about emotion. In this portion of the protocol, unlike the earlier portions that were purposely delivered in a neutral tone, interviewers *emphasized* behavioral emotion cues (i.e., vocal and facial expressions) while using puppets from the AKT to enact stories. In total, eight situations were depicted in which the main character experienced happiness, sadness, anger, or fear. At the end of each story, children were asked to identify how they thought the protagonist felt. Responses were scored in the same way as the other AKT tasks and yielded a summary score for *Behavioral Situational EK* (1 ≤ *x* ≤ 16; α = 0.71). Standardized scores were used in analyses.

#### Externalizing Behavior and Withdrawn Behavior

##### Parent-Report

Children’s parents completed the 100-item Child Behavior Checklist normed for our age group (CBCL 1 ½–5; Achenbach and Rescorla, 2000), which yields two superordinate scales and eight subscales—all of which have displayed high internal consistency (ranging from 0.66 to 0.92), test–retest reliability (ranging from 0.63 to 0.97), and convergent validity with other standardized measures (Achenbach and Rescorla, 2000). Of these, the Externalizing superordinate scale (i.e., “Easily frustrated”; “Defiant”; “Can’t Concentrate”) T scores and the Withdrawn subscale (i.e., “Refuses active games”; “Little Affection”; “Little Interest”; “Withdrawn”) T scores were analyzed.

##### Teacher-Report

Children’s teachers completed the 100-item Child Behavior Checklist-Teacher Report Form (C-TRF; Achenbach and Rescorla, 2000). The magnitude of association between parent and teacher ratings of externalizing behavior and withdrawn behavior was low [*r*(62) = 0.19, *p* = 0.14, *r*(62) = 0.33, *p* < 0.01, respectively]. Moreover, teacher reports of withdrawn behavior were highly positively skewed, and normalcy was not improved with transformations. Therefore, analyses on child externalizing and withdrawn behavior were conducted solely using parent reports.

### Statistical Analysis

#### Power Analysis

To determine the sample size needed to ensure sufficient power (*b* = 0.8; Cohen, 1988), we gathered observed effect sizes for the associations between (a) withdrawn behavior and language and (b) language and EK from the existing literature and pooled them to generate expected effect sizes. From these estimates, we determined that we could expect a medium effect size (0.3 < *r* < 0.5; Cohen, 1988) for the association between withdrawn behavior and language (−0.26 < *r* < −0.41; *M* = −0.35; Milne et al., 2009; Strand et al., 2011; Rudasill et al., 2014) and a large effect size (*r* > 0.5; Cohen, 1988) for the association between language and emotion knowledge (0.45 < *r* < 0.56; *M* = 0.51; Miller et al., 2006; Garner and Waajid, 2008; Morgan et al., 2010; Conte et al., 2019). We then referred to the guidelines provided by Fritz and MacKinnon (2007), which aim to provide empirically derived sample size and power estimates for six different types of mediation analyses based on single sample and resampling simulations. Such guidelines suggested that when a large and medium effect size (LM) condition were expected and analyses utilized percentile bootstrapping, a sample of 59 was sufficient to achieve a power level of 0.8.

#### Missing Data

There were two missing data points for situational EK, one missing for receptive language, 12 missing for withdrawn and externalizing behavior, and 14 missing for CI anger. Participants who were missing reports on externalizing and withdrawn behavior had lower receptive language scores [*t*(71) = −2.49, *p* = 0.02] and lower recognition/expressive EK [*t*(13.07) = −2.38, *p* = 0.02].

#### Main Analyses

To address the first hypothesis that children’s receptive language would mediate the association between withdrawn behavior and EK, we utilized Model 4 of the PROCESS 3.5 macro for SPSS (Hayes, 2018) to conduct mediation analyses for two separate models (i.e., one for recognition/expressive EK and one for situational EK). Mediation was confirmed if the index was significant, and the bias corrected 95% bootstrapped confidence intervals (based on 5,000 bootstrapped samples) did not contain zero. Tertiary to our primary hypotheses, we tested an alternative mediation model with reverse directionality. That is, we tested whether withdrawn behavior mediated the association between receptive language and EK. Rejecting the alternative model would ensure that our proposed mediation model proved to have the best fit for the present data set.

To address the second and third hypotheses that CI anger would positively relate to externalizing and withdrawn behavior, we conducted two separate hierarchical regression analyses. For each regression analysis, we first entered covariates of child age and sex (Step 1), followed by an equation that included both the covariates and CI anger (Step 2).

To address the fourth hypothesis that CI anger would moderate the relationship between language and EK and the fifth hypothesis that CI anger would moderate the mediating effect of language on the association between withdrawn behavior and EK, we conducted a second-stage moderated mediation analysis (Model 14 in PROCESS). First, we determined if the indirect effect of withdrawn behavior on EK significantly varied by conditional level of CI anger. Indirect effects at conditional levels of CI anger were considered significant if the bias corrected 95% bootstrapped confidence intervals did not contain zero. We conducted separate moderated mediation models for recognition/expressive EK and situational EK in instances where corresponding mediation model proved significant.

## Results

Means, standard deviations, and correlations among all variables are reported in Table 1. Correlations between predictors were small to moderate, suggesting multicollinearity was not an issue for the present data set (Cohen et al., 2003). CI anger was positively associated with both externalizing and withdrawn behavior. Greater withdrawn behavior was also associated with lower receptive language. Additionally, withdrawn behavior was found to be negatively associated with situational EK, but it was not associated with recognition/expressive EK. Finally, recognition/expressive EK and situational EK were modestly correlated, and both forms of EK showed the expected positive association with receptive language. Of note, we did not find age or sex differences for most measures. We unexpectedly found younger children had better receptive language, but the association was moderate and mainly attributable to a small number of extreme values in our restrictive age range.

**TABLE 1:** Means, standard deviations, and correlations between predictors and outcome variables.

	1	2	3	4	5	6	7	8	9	*M*	SD
1. CI anger										2.25	1.38
2. Recognition/Expressive EK	0.08									41.65	8.35
3. Situational EK	–0.02	0.40***								12.72	2.72
4. Receptive language	–0.15	0.26*	0.57***							99.88	13.61
5. Withdrawn behavior parent	0.53***	0.05	−0.26*	−0.33**						53.45	5.03
6. Withdrawn behavior teacher	0.29*	–0.07	–0.21	–0.09	0.33**					52.53	5.17
7. Externalizing behavior parent	0.67***	0.18	0.12	–0.04	0.54***	0.26*				47.57	11.30
8. Externalizing behavior teacher	0.15	–0.09	−0.23*	–0.17	0.27*	0.72***	0.19			47.15	10.03
9. Age (months)	0.15	0.17	–0.17	−0.34**	0.25	0.09	0.03	0.18		55.23	5.86
10. Sex	–0.10	0.03	0.15	0.09	–0.11	0.07	–0.06	0.08	–0.04	Female = 51%	

Recognition/Expressive EK was mean of standardized scores for six EK tasks (M, SD are non-standardized sum), situational EK was total score on the AKT puppet task, receptive language was standardized score of PPVT, Withdrawn and Externalizing behavior were T scores on CBCL parent report and C-TRF teacher report. *p < 0.05, **p < 0.01, ***p < 0.001.

### Receptive Language as Mediator Between Withdrawn Behavior and Emotion Knowledge

#### Behavioral Situational Emotion Knowledge

**FIGURE 2:**
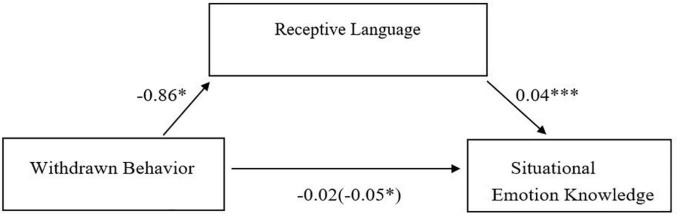
Simple mediation model for the relationship between child withdrawn behavior and situational emotion knowledge, as mediated by receptive language. *p < 0.05, ***p < 0.001.

As indicated in Figure 2, higher withdrawn behavior was associated with lower receptive language, which was associated with lower situational EK. Per hypothesis one, the indirect effect through receptive language was significant (*b* = −0.04, *SE* = 0.02, 95% CI [−0.08, −0.002]) indicating that receptive language acts as a mediator between child withdrawn behavior and situational EK. Although the total effect was significant (*b* = −0.05, *SE* = 0.03, 95% CI [−0.10, −0.003]), the direct effect of withdrawn behavior was not significant (*b* = −0.02, *SE* = 0.02, 95% CI [−0.06, 0.03]). Taken together, about 31% of the variance in situational EK was accounted for when both receptive language and withdrawn behavior were included (*R*^2^ = 0.31). Further, the change in R^2^ associated with receptive language was of considerable magnitude (Δ*R*^2^ = 0.25; Cohen, 1988).

#### Recognition/Expressive Emotion Knowledge

In contrast, receptive language did not mediate the association between withdrawn behavior and recognition/expressive EK (indirect *b* = −0.01, *SE* = 0.01, 95% CI [−0.03, 0.001]). Withdrawn behavior was significantly associated with lower receptive language (*b* = −0.86, *SE* = 0.32, 95% CI [−1.50, −0.22]), but receptive language was not associated with greater recognition/expressive EK (*b* = 0.01, *SE* = 0.01, 95% CI [−0.002, 0.02]). The total effect was not significant (*b* = 0.01, *SE* = 0.02, 95% CI [−0.02, 0.04]), and the direct effect of withdrawn behavior on recognition/expressive EK was not significant (*b* = 0.02, *SE* = 0.02, 95% CI [−0.02, 0.05]).

### Alternative Model: Withdrawal as a Mediator Between Receptive Language and Emotion Knowledge

When we tested the alternative model, withdrawn behavior did not mediate the association between receptive language and situational EK (indirect *b* = 0.002, *SE* = 0.004, 95% CI [−0.004, 0.01]). However, although receptive language was significantly associated with lower withdrawn behavior (*b* = −0.12, *SE* = 0.05, 95% CI [−0.22, −0.03]), lower withdrawn behavior was not significantly associated with greater situational EK (*b* = −0.02, *SE* = 0.02, 95% CI [−0.06, 0.03]). The total effect was significant (*b* = 0.04, *SE* = 0.01, 95% CI [0.03, 0.06]), and the direct effect of receptive language on situational EK was significant (*b* = 0.04, *SE* = 0.01, 95% CI [0.02, 0.06]).

### Associations Between Context-Inappropriate Anger and Externalizing and Withdrawn Behavior

As expected, children who expressed more CI anger were rated by parents as showing more externalizing behavior than other children (see Table 2)—demonstrating support for hypothesis two. Results also supported hypothesis three. That is, children who expressed more CI anger were rated by their parents as showing more withdrawn behavior (see Table 3).

**TABLE 2:** Hierarchical multiple regression on the prediction of externalizing behavior by CI anger.

Step and predictor variable	*R* ^2^	Δ *R*^2^	*sr*	β
Step 1: Covariates	0.01	0.01		
Age			0.10	0.10
Sex			–0.04	–0.04
Step 2:	0.45***	0.43***		
CI anger			0.66***	0.67***

***p < 0.001.

**TABLE 3:** Hierarchical multiple regression on the prediction of withdrawn behavior by CI anger.

Step and predictor variable	*R* ^2^	Δ *R*^2^	*sr*	β
Step 1: Covariates	0.10	0.10		
Age			0.29*	0.29*
Sex			–0.09	–0.08
Step 2:	0.33***	0.23***		
CI anger			0.51***	0.49***

*p < 0.05, ***p < 0.001.

### Context-Inappropriate Anger Moderation of Mediational Model

**FIGURE 3:**
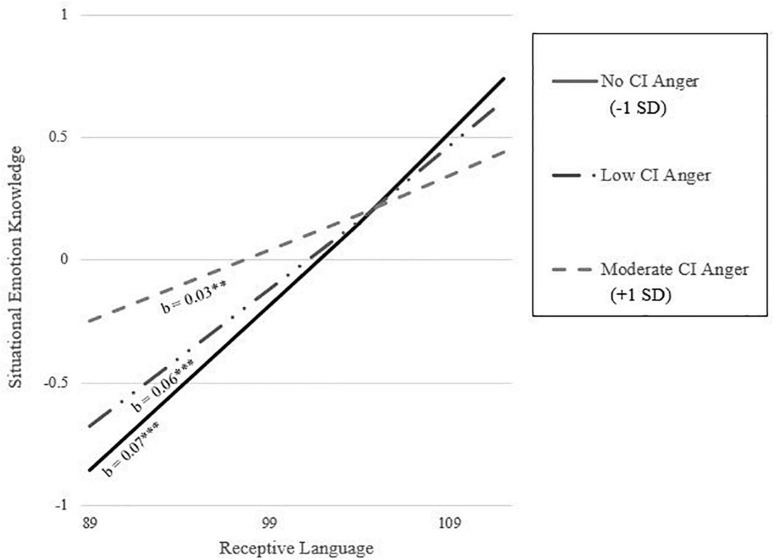
Moderation of association between receptive language and situational emotion knowledge by CI anger. Conditional effects are represented by groupings of the 16th, 50th, and 84th percentile. **p < 0.01, ***p < 0.001.

Per hypothesis four, there was a significant interaction between CI anger and receptive language on situational EK (see Table 4), wherein the positive relationship between receptive language and situational EK was steeper at lower levels of CI anger (see Figure 3). Further, hypothesis five was supported with a significant moderated mediation effect (Index = 0.02, *SE* = 0.01, 95% CI [0.0002, 0.04]). Specifically, there was a significant indirect effect of withdrawn behavior on situational EK via receptive language when CI anger was absent or low but not when CI anger was moderate (see Table 4).

**TABLE 4:** CI anger moderation of language mediation effect on situational EK in withdrawn children.

	Receptive language			
			95% CI	
	*b*	SE	LLCI	ULCI
Predictor variables				
Withdrawn behavior	−0.90**	0.31	–1.52	–0.29
	**Situational EK**			
Withdrawn behavior	–0.02	0.03	–0.08	0.04
Receptive language	0.08***	0.02	0.05	0.12
CI anger	1.78*	0.67	0.44	3.12
CI anger × language	−0.02*	0.01	–0.03	–0.004
Effect of language at No (-1 SD) CI anger	0.07***	0.01	0.04	0.10
Effect of language at low CI anger	0.06***	0.01	0.03	0.08
Effect of language at (+1 SD) moderate CI anger	0.03**	0.01	0.01	0.05
Conditional indirect effects				
CI anger level: None (-1 SD)	–0.06	0.03	–0.13	–0.01
CI anger level: Low	–0.05	0.03	–0.11	–0.004
CI anger level: Moderate (+1 SD)	–0.03	0.02	–0.06	0.004

*p < 0.05, **p < 0.01, ***p < 0.001.

## Discussion

### Language as a Mediator Between Withdrawn Behavior and Emotion Knowledge

Receptive language was found to fully mediate the effect of withdrawn behavior on situational EK. Specifically, greater withdrawn behavior predicted poorer receptive language, and poorer receptive language, in turn, predicted poorer situational EK. These findings support previous studies, which have demonstrated an association between language and EK (e.g., Miller et al., 2006; Morgan et al., 2010). They also expand upon the extant literature in two key ways. Firstly, present findings suggest a direction of effect which differs from most prior research (e.g., Smith-Watts et al., 2014). Previous studies have proposed that language deficits may contribute to withdrawn behavior, but only a few have suggested that withdrawn behavior may lead to language deficits (e.g., Evans, 2010; Strand et al., 2011). Those that have often studied only avoidant/fearful children (e.g., Strand et al., 2011). Secondly, our study findings outline an explicit mechanism by which withdrawn behavior may impact EK through language. Given that peer interaction is cardinal to language development (Kuhl, 2007; Bruce and Hansson, 2011; Curby et al., 2015), and language development is cardinal to EK (e.g., Dunn et al., 1991; Izard et al., 2001), the proposed mechanism is justifiable. Moreover, alternative models with reverse directionality (i.e., language deficits impacting EK through withdrawn behavior), were unsupported by the present data set.

### Behavioral Characteristics of Children Who Show Context-Inappropriate Anger

The positive association between CI anger and externalizing behavior observed in previous studies (Locke et al., 2015, 2017; Locke and Lang, 2016) was replicated. CI anger was also found to be positively associated with withdrawn behavior. Hence, decreased emotional competence associated with CI anger may be a risk factor for co-current behavior problems. Given that co-occurrence of internalizing and externalizing behaviors has been observed in prior studies (Jackson et al., 2000; Verduin and Kendall, 2003), such findings are not unexpected. Furthermore, there may be several plausible explanations for such co-occurrent maladaptive behaviors in children specifically showing CI anger. For instance, CI anger is negatively associated with EK, and poor EK is associated with both externalizing behavior (Dodge et al., 2002; Garner et al., 2008; Trentacosta and Fine, 2010) and withdrawn behavior (Schultz et al., 2001; Trentacosta and Fine, 2010). Additionally, children showing CI anger are more likely to show hypoarousal (Locke et al., 2009), which may simultaneously predict externalizing behavior (e.g., McBurnett et al., 2000) and disengagement with one’s surroundings (Adam and Gunnar, 2001; Gunnar and Vazquez, 2001).

### The Moderating Influence of Context-Inappropriate Anger

Although children who show CI anger may be more likely to withdraw, it appears that how that translates to their ability to learn situational EK goes beyond language deficits. Results from our moderation analysis indicated that increasing levels of CI anger significantly attenuated receptive language’s ability to predict situational EK. Additionally, when incorporated into our moderated mediation model, moderate levels of CI anger rendered the mediating effect of receptive language non-significant. One possible explanation is that there may be fundamentally different reasons why children with CI anger withdraw and, thus, different mechanisms by which withdrawn behavior predicts decrements to situational EK. Several researchers conceptualize withdrawn behavior as a heterogeneous construct (Asendorpf, 1990; Rubin and Asendorpf, 1993). Asendorpf (1990), specifically, distinguishes between three forms of withdrawn behavior based on co-current approach and avoidance motivations: shyness (high approach, high avoidance), unsociability (low approach, low-to-moderate avoidance), and avoidance (low approach, high avoidance). It may be the case, then, that children with moderate or higher levels of CI anger who display withdrawn behaviors are unsociable (i.e., have a non-fearful preference for solitude; Coplan et al., 2019). Consequently, such children may lack motivation to engage in and learn contextual cues related to emotion regardless of language abilities. Conversely, shy children who develop poor EK may do so because their fearfulness drives them to avoid social interactions that would allow them to actively learn language skills necessary for the development of emotional knowledge.

### Study Limitations and Future Directions

Findings from the present study are significant as they both illustrate the intimate interconnections which exist between withdrawal, language, and emotion knowledge and elucidate the nature of CI anger, a relatively unexplored form of emotion dysregulation proposed by Locke et al. (2009). Moreover, our proposed conceptual model was supported, suggesting that there may be several developmental pathways leading to decrements in emotion knowledge. Taken together, these findings may have significant implications for interventions aimed at improving emotion knowledge among preschoolers, as they suggest that for some withdrawn children improving language ability may improve emotion knowledge, while for others alternative factors (e.g., hypocortisolism and resultant low arousal, disengagement, and poor executive function) may need to be addressed. However, though this study and the conceptual model proposed within it do serve to advance the literature in key ways, neither are without limitations.

Firstly, the cross-sectional nature of our design prevents any supposition on temporal precedence. However, as noted in Hayes (2018), the use of mediation analyses should not be considered inappropriate for cross-sectional designs, as inferences about causality are not the product of these mathematical tools but, rather, the product of our interpretation. Consequently, though we must caution readers that there may be other plausible theoretical explanations for our findings, we still advocate that this study and the conceptual model presented within it serve to advance the literature. To improve upon this model further, future research should utilize longitudinal designs and explore additional competing models. The latter would be particularly helpful in clarifying some of the intricacies of how behavioral tendencies impact CI anger (and vice versa). For instance, we found CI anger was associated with withdrawn behavior after controlling for age. This may indicate that temperamental factors associated with CI anger are relevant across preschool ages.

A second limitation concerns the generalizability of our findings. Firstly, we used the PPVT as a proxy for general language comprehension. Although this is in line with other research on emotion knowledge (Cutting and Dunn, 1999; Schultz et al., 2001; Miller et al., 2006), additional research using other language measures would be prudent. Given the commonly observed negative association between childhood withdrawal and both receptive and expressive language, expressive language would be a likely candidate for exploration. Additionally, if vocabulary is integral to forming adequate emotion knowledge, as our study suggests, we advise future research investigate populations with less proficiency, such as those who grow up in bilingual, non-English speaking, or low SES homes (Hua and Wang, 2021). For these children, a clear link between language and social-emotional development has been demonstrated (Washington-Nortey et al., 2022). We would also advocate for a more extensive assessment of the association between emotional competence and CI anger in children growing up with elevated cumulative risk factors in the home, including low SES. Although children living in poverty appear to be more at risk for dysregulated emotion (e.g., Evans, 2003; Raver, 2004), this association may be tempered by factors relevant for CI anger. For example, low-income preschoolers may find it more difficult to regulate anger rather than sadness (Garner and Spears, 2000). Therefore, in studies of early risk factors it would be important to assess regulation of various emotions to capture dysregulation, and we would advocate doing so in various emotional contexts. Our measure of withdrawn behavior may also not generalize beyond parental observation. Knowing parents and teachers observe child behavior in different contexts, we garnered both perspectives. However, most teachers did not endorse withdrawn behavior or focused only on extreme cases. This is not entirely surprising, as other studies have observed internalizing behaviors to be particularly underreported by teachers and affected by biases (Splett et al., 2020; Zee and Rudasill, 2021). This may be because teachers are more aware of externalizing behaviors, given that they are generally more disruptive to the classroom. To combat the limitations of other-rater reports, future research should consider measuring withdrawn behavior through behavioral observation. Finally, our study was limited to children at the older preschool ages, prompting future designs to broaden their scope to include earlier preschool ages that would capture unfolding language and emotion knowledge abilities.

A third limitation of our study is lack of specificity. The present study did not differentiate withdrawn behavior subtypes on the basis of approach and avoidance motivations. Consequently, assertions about differing motives for withdrawn behavior and how they may differentially influence situational emotion knowledge remain speculative. Future research should, thus, aim to elucidate the nature of the moderated mediation effect observed in this study by evaluating withdrawn behavior as a multidimensional construct (i.e., by evaluating each type of withdrawal separately within the context of our conceptual model). Future studies could also include specific aspects of withdrawn behavior (e.g., inhibition and introversion) as covariates within analyses of a broader withdrawal construct to determine if certain aspects of withdrawal are not implicated in poor EK and CI anger. However, to this last point, the existing literature supports the notion that even predominantly inhibited, fearful children tend to have poorer EK than their peers. For instance, studies suggest that inhibited children may misinterpret others emotions, particularly in ways that may promote their fearful responses (Reeb-Sutherland et al., 2015), and those who socially observe do not appear to learn about emotion any better from afar (LaBounty et al., 2017).

Another caveat inherent to not conceptualizing withdrawal as a multidimensional construct is that, while ratings on the CBCL withdrawn subscale can broadly identify a child who behaviorally withdraws from their surroundings, and it commonly serves as an indicator of social withdrawal (e.g., Rubin et al., 2013), it may only be mildly related to fearful withdrawal in children who are behaviorally inhibited (Ballespi et al., 2013). In contrast, it may capture lack of engagement for children who are more unemotional (Benesch et al., 2014). Callous-unemotional traits are more likely seen in aggressive children (Kahn et al., 2012). Consequently, when accompanied by low arousal, greater withdrawal on the CBCL may indicate lack of engagement. It is therefore important to consider other factors in addition to behavioral measures of withdrawal. Taken with our past findings on hypoarousal, our findings would suggest that callous-unemotionality would be a likely candidate for clarifying the greater withdrawn behavior seen in children showing CI anger. Additionally, it may be beneficial to further explore biological correlates (e.g., HPA-axis activity, cardiac regulation, prefrontal cortex-amygdala neurocircuitry) of CI anger in order to improve understanding of the physiology underlying withdrawn behavior in children who show CI anger versus children who do not. Other cognitive and affective factors which may serve to inform our observed moderated mediation should also be investigated. Thus, in addition to exploring physiological correlates and specific withdrawn behavior motivations, additional factors which may reasonably lead to poverty in emotion knowledge should be explored. Considering CI anger’s relationship with externalizing behavior and its shared cortisol profile of attenuated HPA-axis activity (Locke et al., 2009), affective and cognitive factors specific to externalizing behavior may be particularly worth studying.

A fourth limitation surrounds our measurement of emotion knowledge. We propose specific affective and social cognitive factors that could be particularly salient for CI anger given our findings. However, there are specific affective tasks that should be more relevant for CI anger than others. For example, during the non-stereotypical version of the AKT puppet task (Denham, 1986) the child’s atypical emotions are enacted during the vignettes, but only common emotional choices are portrayed in the task (e.g., “happy” or “sad” to come to day care). For future studies in this area, it would be worthwhile to have puppets enact uncommon emotions (e.g., “angry” to come to daycare). Further, rather than measuring overall accuracy in emotion knowledge, it may be important to also consider biased emotion knowledge. For children who show CI anger their anger may stem from misinterpreting others’ intentions as hostile in otherwise ambiguous situations (hostile attribution bias; Dodge and Coie, 1987). This tendency to see more hostility in others may have come about in part because they misread others’ emotional expressions as angry, as seen in more aggressive children (Fine et al., 2004; Garner et al., 2008). Longitudinal studies that capture some of these factors could provide some insight on the nature of CI anger and how it relates to affective processing.

Finally, exploration of our study variables (i.e., withdrawal, CI anger, receptive language, and situational emotion knowledge) in pediatric clinical populations at high risk for poor social functioning is warranted. These may include, but are not limited to, children with externalizing disorders [e.g., attention deficit hyperactivity disorder (ADHD), disruptive disorders] and/or internalizing disorders (e.g., social anxiety, depression), as well as those who commonly present a combination of internalizing and externalizing behaviors [e.g., autism spectrum disorder (ASD)]. These children would be important to consider given the possible deficits in language, emotion knowledge, and emotion regulation associated with these disorders (Philip et al., 2010; Jaffee, 2017; Beauchaine and Crowell, 2020; Northrup et al., 2021). Moreover, evaluating such constructs more broadly across disorders may serve to not only inform our understanding of various developmental trajectories which lead to decreased emotional competence, broader affective social competence, and maladaptive behaviors but may also ultimately serve to support the development of transdiagnostic treatment approaches.

## Conclusion

The present study builds upon our understanding of emotional competence in several ways. Firstly, this study demonstrates that withdrawal and emotional competence are intimately interconnected. Withdrawn behavior was found to negatively predict situational EK. Moreover, findings highlight the central role of language in the effect of withdrawn behavior on situational EK. Though previous studies have suggested withdrawn behavior negatively predicts language and EK, none, to our knowledge, have demonstrated language to be a mediator between withdrawn behavior and EK. Secondly, this study both replicated the association between CI anger and externalizing behavior, as well as uncovered a novel association between CI anger and withdrawn behavior. Finally, findings illuminate the nature of CI anger and its ability to moderate our observed mediation effect. More precisely, CI anger was found to attenuate the effect of language on the relationship between withdrawn behavior and situational EK. As such, this finding suggests that there may be various developmental pathways that lead from early withdrawn behavior to decrements in EK and that factors related to CI anger helps to differentiate these pathways.

## Data Availability Statement

The raw data supporting the conclusions of this article will be made available by the authors, without undue reservation, to any qualified researcher following the investigator’s local IRB guidelines.

## Ethics Statement

The studies involving human participants were reviewed and approved by the University of Massachusetts Dartmouth Institutional Review Board. Written informed consent to participate in this study was provided by the participants’ legal guardian/Parent.

## Author Contributions

RL and RS conceptualized and designed the study. SC, SB, and RL entered, cleaned, and analyzed the resultant data set. SC wrote the first draft of the manuscript. RL, SB, and RS helped to draft the manuscript. All authors contributed to manuscript revision, and read and approved the final version being submitted.

## Conflict of Interest

The authors declare that the research was conducted in the absence of any commercial or financial relationships that could be construed as a potential conflict of interest.

## Publisher’s Note

All claims expressed in this article are solely those of the authors and do not necessarily represent those of their affiliated organizations, or those of the publisher, the editors and the reviewers. Any product that may be evaluated in this article, or claim that may be made by its manufacturer, is not guaranteed or endorsed by the publisher.
